# Music-based immersive technology experience and adolescents’ perceived alleviation of anxiety symptoms: cross-sectional evidence on the mediating roles of cognitive restructuring, self-acceptance, and self-efficacy

**DOI:** 10.3389/fpsyt.2026.1917295

**Published:** 2026-10-19

**Authors:** Ye Ni, Zhaolei Sun, Zhaofeng Li, Enyu Piao, Jianan Zhang

**Affiliations:** 1 Graduate School of Global Convergence, Kangwon National University, Chuncheon, Republic of Korea; 2 School of Music and Dance, Zunyi Normal University, Zunyi, Guizhou, China; 3 School of Music and Dance, Henan Normal University, Xinxiang, China

**Keywords:** adolescents’ perceived anxiety symptoms, cognitive restructuring, music-based immersive technology experience, self-acceptance, self-efficacy

## Abstract

The rising prevalence of anxiety among adolescents, coupled with its long-term negative impact on mental health and well-being, underscores the urgent need to identify and understand key factors that can alleviate these mental health issues. While music experience has been recognized as a promising approach, its integration with immersive technologies remains underexplored. This cross-sectional study examines how music-based immersive technology experience relates to adolescents’ perceived alleviation of anxiety symptoms, focusing on the statistical mediating roles of cognitive restructuring, self-acceptance, and self-efficacy. Data from 457 Chinese adolescents’ self-report survey were analyzed using structural equation modeling. Results indicate that engagement in music-based immersive technology experience is positively associated with the adolescents’ perceived alleviation of anxiety symptoms. In addition, this engagement is positively associated with the cognitive restructuring, self-acceptance, and self-efficacy. These psychological processes are, in turn, positively associated with the perceived alleviation of anxiety symptoms. Mediation analyses show that cognitive restructuring, self-acceptance, and self-efficacy significantly statistically mediate the association between music-based immersive technology experience and the adolescents’ perceived alleviation of anxiety symptoms. Theoretically, this study highlights cognitive restructuring, self-acceptance, and self-efficacy as psychological resources statistically linking immersive music engagement to the adolescents’ perceived alleviation of anxiety symptoms. Practically, the findings suggest that integrating music-based therapeutic principles with immersive technologies may support adolescents’ perceived anxiety-related mental health by being associated with these psychological resources.

## 1 Introduction

Emotional symptoms and disorders, such as depression and anxiety, represent a significant challenge to public health (1). Anxiety remains the most prevalent mental disorder globally, significantly increasing the global disease burden (2). Recent research indicates that the frequency of these symptoms has been rising over the past few decades (3). Studies show that mental disorders are still one of the top ten causes of global burden, with no significant decrease since 1990 (4, 5). In the past decade, the prevalence rate of anxiety disorders has been between 4.34% and 4.58%, with the rates of depression and anxiety among adolescents continuing to rise sharply (6, 7). It is noteworthy that with the intensification of social development and competitive pressures, mental health issues among Chinese children and adolescents are becoming increasingly prominent (8).

Anxiety is a subjective feeling of unease, worry, and concern about future events, which may manifest as a range of physiological and psychological symptoms: irritability, palpitations, heart palpitations, chest tightness, fatigue, cold sweat, etc. (9). Anxiety symptoms in adolescents are associated with functional impairment, academic stress, family factors, emotional distress, and low quality of life (10). Additionally, adolescents with anxiety-related issues often exhibit various physiological, cognitive, and behavioral changes (11), and may be characterized by persistent worry, tension, and excessive future concerns (12). Anxiety symptoms and disorders during this developmental stage have a significant impact on social mental health and increase the likelihood of dropping out of school (13), and may even lead to a burden of disability (14, 15). This indicates that anxiety is not just a mental and psychological health issue; it also affects the quality of life, academic performance, self-perception, life goals, and social functioning of adolescents (16, 17).

Music is an important medium of communication that can transcend emotional barriers and have a positive impact on individuals’ emotional experiences (18, 19). Since ancient times, music has been regarded as a means of supporting the body, emotions, and mind (20). As an organized form of sound, music involves the arrangement of different sounds into structured patterns, such as melody, rhythm, and harmony (21, 22). Music can evoke emotions and prompt people to think in specific ways, which may, in turn, influence their behavior (23).

In the context of mental health, previous studies suggest that music-based approaches may support emotional regulation and symptom relief (24–27). Music therapy and soothing music have been associated with improved mental health outcomes, while art- and music-based audio-visual stimuli have been linked to reduced post-traumatic stress symptoms among adolescents (28–30). Among adolescents, music-based activities may also facilitate emotional expression, attention regulation, and subjective well-being (31). For those who find it difficult to express anxiety-related experiences verbally, music may provide an alternative medium for emotional engagement and self-reflection. However, the present study does not examine music as a stand-alone clinical treatment; instead, music is considered a component of music-based immersive technology experience, a structured context associated with adolescents’ perceived anxiety-related alleviation and related psychological resources.

On the other hand, by combining music-based activities with the immersive multisensory experiences provided by virtual reality (VR) technology (32), MBITE may provide a structured context in which auditory and visual stimuli are presented together. Previous studies have examined immersive technologies in several healthcare and educational contexts, such as cardiology (33), pregnancy (31), oral health (34), and other areas. It is noteworthy that Mitsea, Drigas, and Skianis (35) investigated the value of artificial intelligence, immersive technologies, and neurotechnology in promoting mental and emotional health through respiratory interventions, suggesting that immersive technologies may be relevant to research on emotional health. However, upon reviewing the existing literature, we find that there are few studies focused on anxiety-related perceptions and psychological correlates, especially among adolescents. Therefore, this study aims to address this gap from an exploratory and associational perspective.

Adolescence is a critical period characterized by increased vulnerability to anxiety and other mental health challenges, with long-lasting effects on emotional well-being (10). As such, examining factors associated with adolescents’ perceived anxiety-related experiences is important. Traditional interventions typically focus on clinical treatments aimed at symptom modification. However, this study takes a different approach by exploring music-based immersive technology experience (MBITE) as a non-interventional experiential context associated with adolescents’ perceived anxiety-related outcomes. Rather than directly treating participants’ clinical anxiety, this study examines how engagement with MBITE is statistically associated with perceived alleviation of anxiety symptoms (PAAS), cognitive restructuring (CR), self-acceptance (SA), and self-efficacy (SE). By examining these psychological factors in the context of a six-week MBITE program, this study aims to offer deeper insights into the correlational patterns linking MBITE, psychological resources, and PAAS among adolescents.

On this basis, this study focuses on three theoretically relevant statistical mediators: cognitive restructuring, self-acceptance, and self-efficacy. Cognitive restructuring reflects adolescents’ capacity to reinterpret negative thoughts and stressful experiences in a more adaptive manner; self-acceptance captures a more compassionate and less judgmental attitude toward the self; and self-efficacy represents perceived competence in coping with difficulties and regulating emotions. These three processes correspond to cognitive, self-related, and motivational psychological resources that may statistically account for the association between MBITE and PAAS. Because the study is cross-sectional, these mediating associations should not be interpreted as causal evidence that MBITE causes changes in these psychological resources or that these resources causally produce perceived anxiety alleviation.

Importantly, this study is not designed as an intervention trial and does not include a control group. Instead, it adopts a cross-sectional survey design and uses structural equation modeling to test a theory-driven mediation model among Chinese adolescents. The findings may contribute to the theoretical development of immersive music-based mental health support and provide empirical guidance for future longitudinal and intervention studies. However, the present design does not permit conclusions about therapeutic efficacy, symptom reduction, treatment effectiveness, temporal ordering, or causal mediation.

## 2 Literature review and research hypothesis formulation

### 2.1 Music-based immersive technology experience and perceived alleviation of anxiety symptoms

In recent years, immersive technologies, particularly virtual reality (VR) and augmented reality (AR), have been increasingly integrated into mental health support and intervention contexts related to mental health conditions, including anxiety disorders (36, 37). Music therapy, which has long been utilized to address emotional and psychological challenges, has shown promising results in reducing anxiety levels in various populations, including adolescents (20). Extending this line of research, combining music-based experiences with immersive technologies, such as VR environments or AR applications, can provide a unique multi-sensory experience that enhances the emotional and psychological benefits of music-based engagement (38). This integration allows for a more engaging and personalized approach to mental health support, immersing the individual in a controlled, calming environment where music-based stimuli or activities can be dynamically adjusted based on the participant’s responses. The immersive nature of the technology enables a deepened emotional connection with the music, potentially leading to greater perceived alleviation of anxiety symptoms (39, 40).

The underlying mechanisms by which music-based immersive technology experiences may alleviate perceived anxiety symptoms in adolescents can be understood through several psychological and neurobiological frameworks. The theory of “presence” in virtual environments (41, 42) suggests that immersion in a VR or AR setting fosters a heightened sense of engagement and focus, which could reduce anxiety by distracting the individual from external stressors (43, 44). Additionally, music has been shown to influence the autonomic nervous system (ANS), with specific frequencies and rhythms promoting relaxation and reducing physiological markers of stress, such as heart rate and cortisol levels (45, 46). When coupled with immersive technology, music-based experiences may strengthen these effects by providing an environment that is conducive to relaxation and emotional regulation. Together, these theoretical perspectives provide a robust framework for understanding how music-based immersive technology experience may be associated with adolescents’ perceived alleviation of anxiety symptoms.

Therefore, the following research hypotheses can be proposed:

H1: Music-based immersive technology experience (MBITE) is positively associated with alleviation of perceived alleviation of anxiety symptoms (PAAS) among adolescents.

### 2.2 The mediating role of cognitive restructuring

In recent years, the integration of immersive technologies, such as virtual reality (VR) and augmented reality (AR), with music therapy has emerged as a novel approach for addressing mental health challenges. One of the core mechanisms through which music-based immersive technology experiences may support mental health outcomes is through its potential to promote cognitive restructuring. Cognitive restructuring, a fundamental concept in cognitive-behavioral therapy (CBT), involves the identification and modification of maladaptive thoughts and beliefs that contribute to emotional distress (47). Music-based immersive technology experience, by providing a multi-sensory experience, enhances emotional engagement and creates a novel context in which adolescents can confront and reframe negative thought patterns in a more controlled, immersive environment (3). This process aligns with theories of cognitive-emotional integration, which suggest that immersive environments can facilitate a more immediate and profound reappraisal of distressing thoughts and feelings.

The use of immersive technology in conjunction with music-based experiences capitalizes on the ability to evoke deep emotional responses, which are key to triggering cognitive restructuring. According to the “Dual Process Theory” of cognitive processing (48, 49), individuals engage in both intuitive and reflective thinking when confronted with emotionally charged situations. Immersive experiences have the capacity to stimulate intuitive, emotionally driven responses (50), while the structured, deliberate nature of music-based activities helps guide the adolescent toward reflective thinking. By merging these elements, music-based immersive technology experience provides a dynamic framework for adolescents to both experience their emotions in a controlled space and cognitively challenge the underlying negative beliefs that fuel their anxiety. In doing so, it supports the development of healthier cognitive frameworks that may contribute to the alleviation of anxiety symptoms and foster resilience.

Therefore, the following research hypothesis can be proposed:

H2: Music-based immersive technology experience (MBITE) is positively associated with cognitive restructuring (CR) among adolescents.

Cognitive restructuring, a core component of cognitive-behavioral therapy (CBT), has been widely recognized for its efficacy in alleviating anxiety symptoms, particularly in adolescents (51). According to cognitive theory of anxiety (52, 53), maladaptive thought patterns, such as catastrophic thinking and distorted self-perceptions, play a central role in the development and maintenance of anxiety disorders. By identifying and challenging these cognitive distortions, cognitive restructuring helps individuals reframe negative thoughts, which can subsequently lead to emotional regulation and a reduction in anxiety levels (54, 55). The process of cognitive restructuring enables adolescents to reinterpret anxiety-provoking situations from a more balanced and realistic perspective, reducing the physiological and psychological arousal associated with anxiety (56, 57). This theoretical framework underpins the effectiveness of cognitive restructuring in treating anxiety by addressing the cognitive vulnerabilities that contribute to the disorder’s onset and persistence.

Furthermore, research supports the notion that cognitive restructuring techniques are particularly effective in the adolescent population, where cognitive development is still in progress (58). Adolescents are more prone to experiencing anxiety due to heightened sensitivity to social evaluation, identity formation challenges, and peer pressure (59). Studies have shown that cognitive restructuring, through its emphasis on altering negative thought patterns, can help adolescents develop healthier cognitive schemas that promote resilience against stress and anxiety (60, 61). Thus, integrating cognitive restructuring within a therapeutic framework provides adolescents with the tools to not only manage but also prevent the exacerbation of anxiety, which is particularly beneficial when combined with complementary techniques such as immersive technology-based experiences.

Therefore, the following research hypotheses can be proposed:

H3: Cognitive restructuring (CR) is positively associated with perceived alleviation of anxiety symptoms (PAAS) among adolescents.

Based on these, the following research hypotheses can be inferred:

H4: Cognitive restructuring (CR) significantly mediates the association between music-based immersive technology experience (MBITE) and perceived alleviation of anxiety symptoms (PAAS) among adolescents.

### 2.3 The mediating role of self-acceptance

Music-based immersive technology experiences may promote the development of self-acceptance in adolescents through several interconnected mechanisms that leverage both the emotional power of music and the immersive qualities of technology. First, the immersive nature of virtual or augmented reality (VR/AR) provides a unique environment where adolescents can engage deeply with their emotions in a controlled, non-judgmental space (39). This immersive experience allows them to step outside of the pressures of everyday social interactions and focus on their internal emotional states (36, 62). As adolescents listen to or interact with music in these environments, they can engage in a process of emotional release and regulation, which is crucial for alleviating anxiety and reducing negative self-judgments. This direct engagement with emotion helps them develop greater emotional resilience, which in turn supports self-acceptance by fostering a positive relationship with their feelings and reducing emotional repression.

Second, music-based immersive technology experience may enhance self-reflection through embodied experiences that encourage adolescents to confront and understand their own identity. Self-acceptance is fundamentally tied to the coherence between an individual’s ideal self and real self, as proposed by Carl Rogers’ theory of self-concept (63, 64). By participating in music-based immersive experiences, adolescents are encouraged to explore their self-image through music and visual elements that prompt introspection and self-awareness. For example, VR environments can provide immersive scenarios where adolescents visualize their emotions or confront difficult memories (65), while music acts as a tool to guide these processes. These multisensory experiences create opportunities for adolescents to reconsider their self-perceptions, challenge distorted self-images, and come to terms with their true identity, thus improving self-acceptance. The cathartic and transformative nature of music in these settings allows adolescents to see themselves more clearly, ultimately leading to greater self-compassion and self-approval.

Finally, music-based immersive technology experiences may activate the brain’s reward and emotional regulation systems, particularly those associated with empathy and self-awareness (66, 67). Music itself is known to activate the brain’s reward pathways (68), and when combined with the immersive experience of VR or AR, these effects are heightened. This increased emotional engagement promotes a deeper connection with the self, allowing adolescents to identify and address negative emotional patterns that hinder self-acceptance. Additionally, the interactive aspect of immersive technology enables adolescents to take an active role in their emotional journey (69, 70), making choices and interacting with their immersive environment. This sense of agency reinforces their intrinsic motivation to grow and improve, as supported by self-determination theory. By giving them control over their emotional experience, music-based immersive technology experience may encourage adolescents to embrace their authentic selves, fostering greater acceptance of both their strengths and weaknesses.

Therefore, the following research hypothesis can be proposed:

H5: Music-based immersive technology experience (MBITE) is positively associated with self-acceptance (SA) among adolescents.

Self-acceptance plays a crucial role in alleviating anxiety symptoms in adolescents, as it directly influences their emotional and psychological well-being. According to the theory of self-acceptance (71), individuals experience anxiety when there is a discrepancy between their ideal self and their actual self. Adolescents, in particular, are highly susceptible to such incongruences due to the developmental changes they undergo, which can lead to feelings of inadequacy and self-doubt. By fostering self-acceptance, adolescents can develop a more positive and coherent self-concept, thereby reducing the internal conflict that often manifests as anxiety (72, 73). Self-acceptance involves recognizing and embracing one’s strengths, weaknesses, and inherent worth, which helps to mitigate the emotional distress that accompanies a critical self-view (74, 75).

In the context of music-based experiences, self-acceptance can be enhanced by providing adolescents with opportunities to engage in reflective and expressive activities, which promote a sense of agency and autonomy. Music, as a supportive medium, has been shown to facilitate self-expression and emotional regulation (76). Through immersive and personalized music experiences, adolescents can explore and process their emotions in a non-threatening manner, which supports the development of a more accepting attitude toward themselves. This aligns with the principles of cognitive-behavioral therapy (77), where individuals are encouraged to challenge negative self-beliefs and cultivate healthier self-views. As adolescents develop greater self-acceptance, they are more likely to experience reduced anxiety, as their internal dialogue shifts from self-criticism to self-compassion, fostering emotional resilience and psychological stability.

Furthermore, self-acceptance is linked to the reduction of anxiety through its impact on emotional regulation (78). According to the process model of emotion regulation (79), individuals who accept their emotional experiences are better equipped to manage and modulate their emotions effectively. Adolescents who embrace their emotions, rather than suppressing or denying them, can develop healthier coping strategies for managing anxiety. Music-based experiences, particularly when combined with mindfulness techniques, can support adolescents in cultivating emotional awareness and acceptance, ultimately promoting emotional regulation and reducing anxiety (80). This is particularly important in adolescence, as it is a critical period for the development of coping mechanisms that can last into adulthood. By enhancing self-acceptance through music-based experiences, adolescents can develop more adaptive emotional responses, thereby decreasing anxiety symptoms.

Therefore, the following research hypotheses can be proposed:

H6: Self-acceptance (SA) is positively associated with perceived alleviation of anxiety symptoms (PAAS) among adolescents.

Based on these, the following research hypotheses can be inferred:

H7: Self-acceptance (SA) significantly mediates the association between music-based immersive technology experience (MBITE) and perceived alleviation of anxiety symptoms (PAAS) among adolescents.

### 2.4 The mediating role of self-efficacy

Music-based immersive technology experiences may facilitate the development of self-efficacy in adolescents through mechanisms related to self-reflection and goal achievement. Unlike traditional therapeutic approaches, music-based immersive technology experience creates a dynamic, multisensory environment that encourages active participation (81, 82). In this space, adolescents are not merely passive recipients of supportive experiences but are actively engaged in shaping their immersive experience. This engagement encourages a sense of agency, as adolescents can make decisions within the virtual world and see the immediate consequences of their actions. The ability to manipulate the virtual environment and music elements gives adolescents a sense of autonomy, which strengthens their belief in their ability to influence outcomes in real life (83). This sense of control directly supports the development of self-efficacy by reinforcing the notion that their actions can lead to positive changes.

Another key mechanism involves self-reflection facilitated by music-based immersive technology experience (84, 85). Within the virtual environment, adolescents are presented with scenarios and experiences that are designed to evoke emotional responses. Music, as an emotional trigger, works in conjunction with immersive elements to help adolescents explore their emotions in a deeper and more meaningful way. The real-time feedback provided by the virtual environment allows adolescents to reflect on their emotional responses, recognize patterns, and gain insight into their behaviors. This process of self-reflection, coupled with the opportunity for incremental success within the immersive setting, fosters a stronger sense of mastery. Each successful navigation of emotional or stress-inducing situations within the virtual world contributes to a growing belief in their ability to cope with similar challenges in real life. By framing immersive experiences within a goal-oriented structure—such as overcoming anxiety in a virtual environment—adolescents experience a sense of accomplishment, reinforcing their self-efficacy beliefs and motivating them to apply these strategies outside the immersive context (86, 87). Thus, music-based immersive technology experience may enhance self-efficacy not only through emotional regulation and coping but also through the cultivation of reflective practices and goal-setting experiences that support adolescent empowerment.

Therefore, the following research hypothesis can be proposed:

H8: Music-based immersive technology experience (MBITE) is positively associated with self-efficacy (SE) among adolescents.

Self-efficacy (88) refers to an individual’s belief in their ability to successfully perform tasks and manage challenges. This belief plays a crucial role in alleviating anxiety, particularly among adolescents. Anxiety often arises from the perception of being unable to cope with stressors, leading to feelings of helplessness and avoidance behaviors (89). When adolescents possess high self-efficacy, they are more likely to approach challenging situations with confidence, believing they have the resources to manage their emotions and reactions (90, 91). As a result, they are less prone to experiencing the paralyzing fear that typically accompanies anxiety. The core mechanism through which self-efficacy mitigates anxiety lies in its ability to promote adaptive coping strategies and enhance perceived control over emotional experiences. Adolescents with higher self-efficacy are more inclined to use active coping mechanisms, such as problem-solving or seeking support, instead of resorting to maladaptive responses like avoidance or rumination (92, 93). This proactive approach leads to a reduction in anxiety, as adolescents feel more capable of managing and overcoming stressors, thus breaking the cycle of avoidance and negative emotional reinforcement.

Therefore, the following research hypotheses can be proposed:

H9: Self-efficacy (SE) is positively associated with perceived alleviation of anxiety symptoms (PAAS) among adolescents.

Based on these, the following research hypotheses can be inferred:

H10: Self-efficacy (SE) significantly mediates the association between music-based immersive technology experience (MBITE) and perceived alleviation of anxiety symptoms (PAAS) among adolescents.

## 3 Methodology

### 3.1 Observational study design

Unlike traditional intervention studies that aim to treat clinical symptoms, this research was designed as an observational, non-interventional, and exploratory study. Its purpose was to examine how adolescents’ music-based immersive technology experience (MBITE) was associated with adolescents’ perceived alleviation of anxiety symptoms (PAAS) and related psychological factors. Rather than evaluating the clinical effectiveness of an intervention, the study focused on identifying the associations between MBITE and adolescents’ cognitive and affective self-regulatory resources, including cognitive restructuring (CR), self-acceptance (SA), and self-efficacy (SE). Accordingly, MBITE was treated as an experiential exposure measured in relation to self-reported perceptions, not as a treatment condition designed to test symptom reduction.

This study was conducted in the context of a six-week immersive music experience. During this period, participants engaged in structured music-based sessions supported by immersive technology. The design focused on assessing adolescents’ perceptions and psychological responses related to MBITE, PAAS, CR, SA, and SE. By examining these variables in the context of a six-week music experience, the study aimed to provide insights into the psychological processes that may statistically account for the association between MBITE and PAAS among adolescents.

The temporal sequence of the study procedures is summarized in **Figure 1**. First, schools and classes were selected, and eligible adolescents were invited to participate. Second, written informed consent from parents or legal guardians and assent from adolescents were obtained. Third, the Screen for Child Anxiety Related Emotional Disorders (SCARED) was administered before the six-week MBITE experience, during the recruitment and eligibility-confirmation stage. Fourth, eligible participants completed the six-week MBITE experience. Fifth, immediately after the final MBITE session, participants completed the self-report questionnaire assessing MBITE, CR, SA, SE, and PAAS. Thus, SCARED was administered before the experiential period, whereas the main study variables included in the structural equation model were collected at a single time point after the six-week MBITE experience.

**Figure 1:**
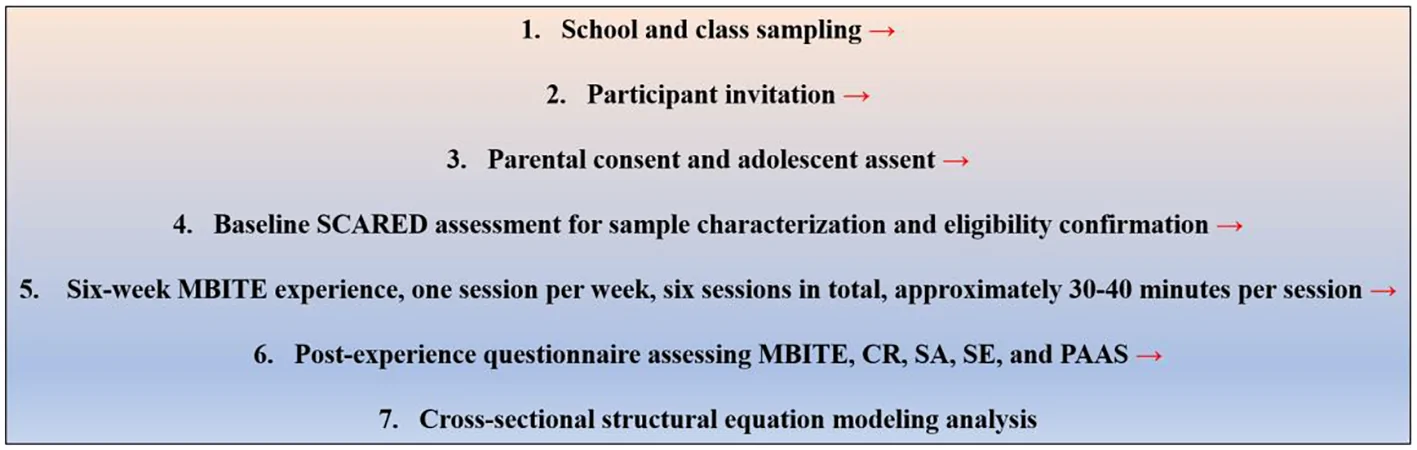
Timeline of study procedures.

The MBITE experience spanned six consecutive weeks. Participants attended one structured session per week, and each session lasted approximately 30 to 40 minutes. Therefore, the planned exposure consisted of six sessions in total for each participant. Attendance was recorded by trained research assistants at each session. Participants included in the final analytic sample completed all six scheduled MBITE sessions and the post-experience questionnaire. Participants who missed one or more sessions, withdrew from the study, experienced VR-related discomfort that prevented continued participation, or provided incomplete post-experience questionnaire data were excluded from the final analysis, as detailed in the participant recruitment and screening flow. Sessions were conducted in a quiet and controlled environment to reduce external distractions and support participants’ engagement with the music experience. Each session incorporated calming and emotionally supportive music delivered through immersive virtual reality (VR) headsets that synchronized visual and auditory stimuli. The immersive environment included carefully curated 360° visual materials, such as natural landscapes, slow-moving abstract patterns, and interactive elements that responded to the music, in order to support attentional focus, emotional presence, and embodied involvement.

The immersive sessions were delivered using PICO Neo 3 Pro standalone VR head-mounted displays. The headset provides a stereoscopic 360-degree visual environment, with a display resolution of 3664 × 1920 pixels, a refresh rate of up to 90 Hz, and an approximate field of view of 98 degrees. Audio was delivered through over-ear headphones connected to the VR headset to maintain consistent sound quality and reduce environmental noise. The immersive materials were developed and presented in a Unity-based software environment, which synchronized preselected 360-degree visual scenes, Chinese traditional music tracks, and short guided verbal prompts. The software standardized the order, duration, and presentation of each session across participants. Before each session, the research team checked headset fit, battery level, audio volume, visual clarity, and participant comfort.

For reproducibility, the MBITE protocol followed a standardized session structure. Each session consisted of five components: a brief orientation and comfort check, an opening breathing and attentional-settling segment, the main immersive music-and-visual experience, short guided verbal prompts embedded in the audio stream, and a closing transition period. The approximate duration of each component was as follows: orientation and equipment check, 3–5 minutes; opening settling segment, 3–5 minutes; main immersive music experience, 20–25 minutes; guided reflection prompts, 3–5 minutes distributed across the session; and closing transition, 2–3 minutes. Participants were instructed to remain seated, listen attentively, observe the virtual environment, and respond internally to the prompts without speaking aloud. No task performance, competitive interaction, or clinical counseling was included during the sessions.

Across the six weeks, the thematic content varied by session to maintain engagement while preserving the same overall structure, duration, and level of stimulation. The music, visual scenes, presentation order, prompt timing, and session structure were fixed for each weekly session. The Unity-based software ensured that all participants received the same audiovisual sequence for a given session. Participant interaction was intentionally minimal: participants could visually explore the 360° environment through natural head movement, but they were not required to make choices, complete tasks, or manipulate virtual objects. This design was used to reduce variability in exposure and to avoid performance-related stress. A detailed session-by-session MBITE protocol is provided in **Supplementary Appendix A**.

The visual scenes were selected to avoid highly stimulating or distressing content. Scenes involving rapid camera movement, flashing lights, sudden transitions, intense colors, or threat-related imagery were excluded to reduce the likelihood of cybersickness, visual fatigue, or emotional discomfort. Participants were informed that they could pause or stop the session at any time if they experienced dizziness, discomfort, distress, or unwillingness to continue.

To enhance cognitive and emotional engagement, the VR-based music sessions also included short guided verbal prompts embedded in the audio stream. These prompts encouraged participants to engage in self-reflection, cognitive reframing, and emotional awareness. The guided prompts were brief, non-diagnostic, and non-therapeutic in wording. They were designed to encourage calm attention, emotional awareness, and reflective appraisal rather than to provide psychotherapy. Examples of prompts included: “Notice your breathing and let your attention settle with the music”; “Observe any worrying thought as it comes and goes”; “Try to look at one difficulty from another angle”; “Allow yourself to accept the present moment without judging yourself”; and “Recall one small challenge you have managed before.” The complete wording and timing of the prompts are reported in **Supplementary Appendix A**. This immersive format was not intended to function as a clinical treatment, but rather as a multisensory music-based experience through which adolescents could engage with emotional expression, regulation, and meaning-making. In this way, this study examined whether engagement with MBITE was associated with PAAS and whether CR, SA, and SE statistically mediated this association.

### 3.2 Cultural specificity and use of Chinese music

A key feature of this study is the use of culturally relevant Chinese music, including guqin, erhu, and bamboo flute. This approach is grounded in music cultural psychology and is intended to enhance emotional resonance and engagement among adolescent participants. Previous research suggests that culturally congruent music may strengthen emotional engagement and cognitive receptivity, providing a rationale for its use in the present study context.

Traditional Chinese music, with its distinctive timbres, rhythms, and melodic structures, may provide a culturally familiar and emotionally meaningful listening experience for Chinese adolescents. Musical characteristics such as the pentatonic scale, slow tempo, and flowing melodies are often associated with relaxation, attentional focus, and meditative states. In the present study, these musical features were used to create a calming and culturally meaningful environment in which adolescents could engage with the immersive music experience. Rather than assuming direct clinical effects, the incorporation of Chinese music was intended to enhance cultural relevance, participant engagement, and ecological validity.

To align the music with the study context, the research team employed a systematic selection process. Participants were invited to provide feedback on the music choices, and this feedback informed the final selection of musical materials. This process emphasized the importance of music preference and perceived cultural familiarity in supporting adolescents’ involvement in the six-week immersive music experience.

Participant feedback on music preference and perceived cultural familiarity was collected before the formal six-week MBITE program began. Specifically, feedback was obtained during a pilot/pretesting stage from 32 adolescents who were comparable in age and school background to the main study participants but were not included in the final analytic sample. These adolescents listened to candidate Chinese traditional music excerpts and rated their perceived familiarity, emotional comfort, calmness, and suitability for an immersive experience. The research team used this feedback, together with expert review by specialists in psychology, music therapy, and immersive technology, to finalize the musical materials before the main data collection. No music tracks, visual scenes, guided prompts, or session sequences were modified after the six-week MBITE program had begun for the analytic sample.

Additionally, this study integrated immersive virtual reality (VR) technology with the selected music to further support emotional engagement and attentional immersion. The VR environments were designed to complement the musical materials and to provide a multisensory context associated with relaxation, mindfulness, and emotional awareness. For example, during erhu-based sessions, VR visuals of expansive landscapes and gentle movements were synchronized with the music to create a calm and reflective atmosphere. These multisensory experiences were intended to support emotional presence, self-reflection, and cognitive engagement, which may be related to cognitive restructuring, self-acceptance, and self-efficacy.

By combining culturally relevant Chinese music with immersive VR technology, this study provides an ecologically meaningful context for examining adolescents’ music-based immersive technology experience. Rather than evaluating the clinical efficacy of a psychological intervention, the study explores how this culturally adapted immersive music experience is associated with perceived alleviation of anxiety symptoms (PAAS) and related psychological factors among adolescents.

### 3.3 Measurement scale design

In this study, the measurement scales were designed to assess adolescents’ music-based immersive technology experience (MBITE) and related psychological variables, including cognitive restructuring (CR), self-acceptance (SA), self-efficacy (SE), and perceived alleviation of anxiety symptoms (PAAS). The scale development process followed a rigorous and systematic procedure to enhance the validity and reliability of the measurement instruments. The measurement items were adapted from established and validated scales that have been widely used in previous research on anxiety-related experiences, cognitive restructuring, self-acceptance, and self-efficacy. These original scales provided a foundational framework, while appropriate modifications were made to ensure their relevance to the specific context of the present study, particularly the integration of immersive technology with music-based experiences. This approach is commonly used in questionnaire-based psychological research when multiple constructs are assessed in one survey, as it helps balance construct coverage, feasibility, and respondent burden. The final item selection was guided by the constructs specified in the study’s hypothesized model, rather than by the development of new independent constructs.

Because each core construct was measured using three selected items, the item adaptation procedure was designed to prioritize conceptual precision, developmental appropriateness, and respondent feasibility. The research team first reviewed the source instruments and identified candidate items that most directly corresponded to the theoretical definition of each construct in the present model. Items were retained or adapted when they met four criteria: first, the item represented the central meaning of the target construct; second, the wording could be understood by adolescents aged 10–18 years; third, the item could be meaningfully answered in relation to the MBITE context; and fourth, the item did not introduce clinical diagnostic wording or imply treatment efficacy. Items that were redundant, overly abstract, clinically worded, or weakly connected to the immersive music context were not retained. No changes were made to the substantive meaning of the final measurement items after data collection.

In this study, the dependent construct was conceptualized as perceived alleviation of anxiety symptoms (PAAS). This construct refers to adolescents’ subjective appraisal that anxiety-related experiences had been reduced or relieved in the context of their engagement with the music-based immersive technology experience. It was not intended to measure clinical anxiety severity, diagnostic status, or objectively verified symptom reduction. Instead, PAAS captures perceived relief in three anxiety-related domains: physical discomfort, repetitive or intrusive anxious thoughts, and emotional uneasiness.

Expert input was solicited at multiple stages of the scale adaptation process. A panel of experts in psychology, music therapy, and immersive technology was invited to review the adapted items and provide feedback on content validity. Their feedback informed further refinements to ensure that each item accurately reflected the construct it was intended to measure while remaining appropriate for the cultural and developmental characteristics of adolescent participants. Expert review also helped ensure that the wording of the items was accessible, clear, and comprehensible to the target population.

Specifically, the expert review panel consisted of six reviewers: two researchers in adolescent psychology and mental health, one researcher in psychometrics and questionnaire design, one music therapy specialist, one immersive technology/VR design specialist, and one secondary-school mental health educator. Each reviewer independently evaluated the candidate items in terms of construct relevance, clarity, developmental appropriateness, cultural appropriateness, and contextual fit with the MBITE experience. Reviewers were asked to identify ambiguous wording, double-barreled expressions, age-inappropriate phrasing, and any item that could be misinterpreted as a clinical diagnosis or treatment claim. Items were revised before the main survey when at least two reviewers raised similar concerns or when a reviewer identified a substantive problem related to adolescent comprehension, cultural meaning, or construct validity. Disagreements were resolved through discussion among the research team until consensus was reached.

The questionnaire was developed and administered in Chinese. For items adapted from English-language source instruments, a translation and cultural adaptation procedure was used before the main survey. First, two bilingual researchers independently translated the candidate items into Chinese. Second, the research team compared the two Chinese versions and produced a reconciled version. Third, a separate bilingual researcher back-translated the reconciled Chinese items into English. Fourth, discrepancies between the back-translated version and the original conceptual meaning were reviewed and resolved. Fifth, the Chinese wording was further adjusted to ensure that the items were natural, concise, and understandable for adolescents in Chinese school settings. The English items reported in **Table 1** are translated reporting versions of the Chinese questionnaire actually administered to participants and do not represent *post hoc* changes to the measurement indicators.

**Table 1:** Study scale and measurement items.

Variables	Measurement	Sources
Music-Based Immersive Technology Experience (MBITE)	MBITE1: When engaging in a music-based immersive technology experience, I experienced a sense of immersion, as if I were in another space.	(94, 95)
	MBITE2: When engaging in a music-based immersive technology experience, I felt a close connection among myself, the music, and the virtual environment.	
	MBITE3: When engaging in a music-based immersive technology experience, I often became deeply absorbed in the experience.	
Cognitive Restructuring (CR)	CR1: I am able to consider the issues that trouble me from different perspectives.	(96, 97)
	CR2: When encountering challenging situations, I can adjust my perspective to reduce negative emotions.	
	CR3: I am able to identify positive aspects of situations even when they are difficult.	
Self-acceptance (SA)	SA1: I am gradually learning to accept my own flaws.	(98, 99)
	SA2: I am able to appreciate the unique aspects of myself.	
	SA3: I can view my shortcomings with a forgiving attitude.	
Self-efficacy (SE)	SE1: I believe in my ability to face various challenges in life.	(100, 101)
	SE2: Even when faced with difficulties, I have confidence in my ability to persevere.	
	SE3: When problems arise, I believe I can make appropriate decisions.	
Perceived Alleviation of Anxiety Symptoms (PAAS)	PAAS1: I perceive a reduction in anxiety-related physical discomfort, such as rapid heartbeat, breathing discomfort, or bodily tension.	(11, 102, 103)
	PAAS2: I perceive a reduction in repetitive or intrusive anxious thoughts.	
	PAAS3: I perceive a reduction in emotional uneasiness related to anxiety in daily life.	

The final version of the measurement scales was pre-tested with a small sample of adolescents to assess item clarity, comprehensibility, and feasibility. Based on the pre-test results, minor revisions were made to improve the wording and structure of several items, thereby enhancing their clarity and contextual relevance. In addition, the internal consistency of each scale was assessed using Cronbach’s alpha. The results indicated satisfactory reliability coefficients for all scales, suggesting that the adapted measurement instruments demonstrated acceptable internal consistency.

The pre-test was conducted with 32 adolescents aged 10–18 years from schools comparable to those in the main study. These adolescents were not included in the final analytic sample. The pre-test focused on item comprehension, response burden, administration time, and whether participants could understand the meaning of each construct in the MBITE context. Participants were asked to indicate whether any item was difficult to understand, repetitive, emotionally uncomfortable, or unclear. The average completion time was approximately 6–8 minutes. No item was removed after the pre-test. Minor wording refinements were made before the main survey to improve readability and avoid clinical terminology, but these refinements did not alter the substantive meaning of the final measurement indicators.

Overall, the measurement scale design in this study involved the adaptation of validated instruments, expert review, and pre-testing. This multi-step process provided a sound measurement basis for examining the associations among MBITE, CR, SA, SE, and PAAS among adolescents, rather than evaluating the clinical effectiveness of an intervention.

Guided by the hypothesized model presented in **Figure 2**, the research team developed three measurement items for each of the five core variables. The selection of these items was based on three considerations: first, they had the strongest conceptual alignment with the original validated scales; second, they captured the core meaning of each construct within the limited length of a cross-sectional survey; and third, they were understandable and appropriate for adolescent respondents in the music-based immersive technology context. These items as shown in **Table 1**. All items were measured using a five-point Likert scale, ranging from 1 = Strongly Disagree to 5 = Strongly Agree, allowing participants to report their perceptions and experiences in a standardized manner. Higher scores indicated higher levels of the corresponding construct. Scale scores were calculated by averaging the three items for each construct, with no reverse-coded items. In addition, because the questionnaire was administered to Chinese participants, the English wording of the measurement items reported in this manuscript represents a translated reporting version. The refinement of the English wording does not alter the substantive meaning of the items, the original data, or any statistical analyses. The complete Chinese-language items actually administered to participants, together with scoring instructions, are provided in **Supplementary Appendix B**.

**Figure 2:**
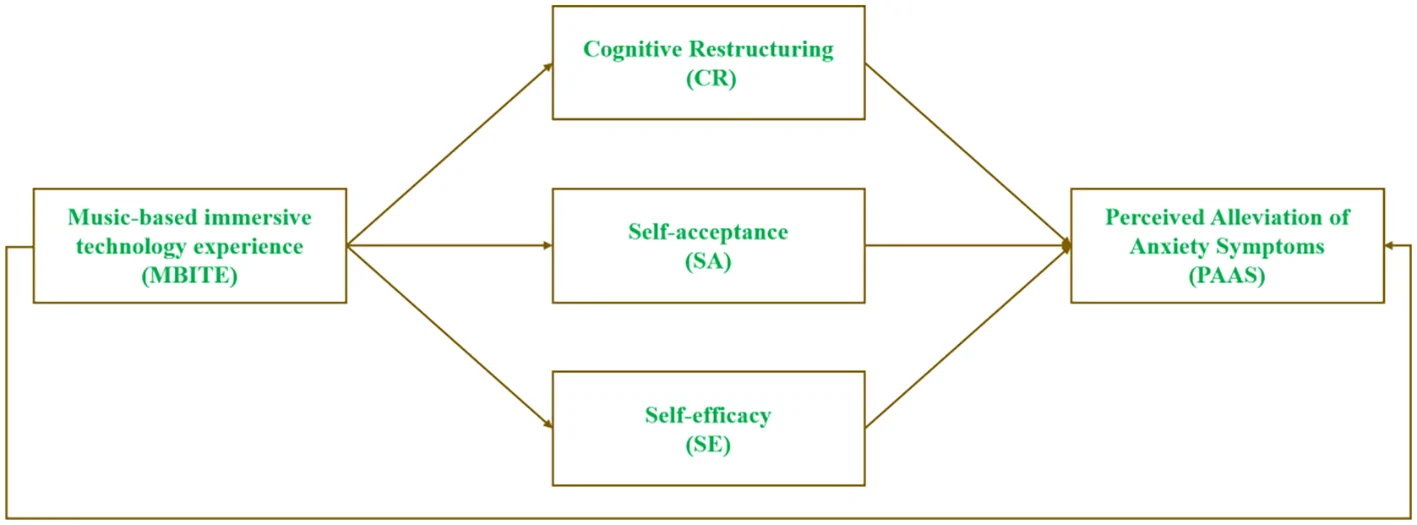
Research hypothesis model of this study.

### 3.4 Participants and sample selection

The sampling frame consisted of full-time students aged 10–18 years enrolled in participating primary, junior high, and high schools in three cities in Eastern China. A multistage stratified sampling procedure was used. First, schools were stratified by city and school type, namely public or private. Second, within each selected school, classes were stratified by grade level. Third, classes within each stratum were selected using a random-number procedure, and all eligible students in the selected classes were invited to participate. Socioeconomic background was considered at the school catchment and family levels, based on available school-level information and parent-reported background information collected during recruitment. This procedure was used to improve demographic coverage and reduce overrepresentation of any single city, school type, or grade group.

Participants were eligible for inclusion if they were enrolled full-time in school, were within the specified age range, were able to understand the study procedures, and could engage with immersive music and digital media technology during the six-week music experience. Participants also reported varying levels of perceived anxiety-related symptoms, as assessed by the Screen for Child Anxiety Related Emotional Disorders (SCARED), a well-validated instrument for adolescent populations. The Chinese child self-report version of the SCARED was administered before the six-week MBITE experience, during the recruitment and baseline characterization stage. The scale contains 41 items rated on a 3-point scale from 0 = not true or hardly ever true to 2 = very true or often true. Total scores range from 0 to 82, with higher scores indicating greater anxiety-related symptom burden. In the present study, the SCARED was used to characterize anxiety-related experiences in the school-based sample and to confirm variability in self-reported symptoms. It was not used to establish a psychiatric diagnosis, was not repeated after the MBITE experience, and was not included as the dependent variable in the SEM analysis. The commonly referenced cutoff score of 25 was used only for descriptive purposes to indicate elevated anxiety-related symptoms. All participants provided informed assent, and written informed consent was obtained from their parents or legal guardians.

Adolescents with clinically diagnosed psychiatric disorders or those currently receiving psychological or psychiatric treatment were excluded to minimize potential confounding effects related to clinical care. Clinically diagnosed psychiatric disorders were identified based on parent/guardian report and school health or counseling records available to the school at the time of recruitment. Parents or guardians were asked whether the adolescent had ever received a formal psychiatric diagnosis from a qualified medical or mental health professional, and whether the adolescent was currently receiving psychiatric medication, psychotherapy, counseling for a diagnosed disorder, or other formal psychological/psychiatric treatment. Students with parent-reported or school-recorded diagnoses, including anxiety disorders, depressive disorders, attention-deficit/hyperactivity disorder, autism spectrum disorder, psychotic disorders, bipolar disorder, or other clinically diagnosed psychiatric conditions, were excluded. The research team did not conduct independent clinical diagnostic interviews; therefore, the exclusion criterion should be interpreted as exclusion based on reported or documented clinical diagnosis rather than researcher-administered diagnosis. Importantly, this study did not recruit clinically diagnosed patients. Instead, it focused on adolescents across a natural continuum of self-reported anxiety-related experiences within school settings.

Participant recruitment and screening flow is summarized in **Table 2**. A total of 560 adolescents were initially approached from selected schools and classes, and all 560 were assessed for eligibility during the recruitment stage. Sixty-eight adolescents were excluded before enrollment, including 28 who did not meet the age or enrollment criteria, 16 who had a parent-reported or school-recorded psychiatric diagnosis or were currently receiving treatment, and 24 who declined participation or had no parental consent. A total of 492 adolescents provided assent and parental consent and were enrolled in the six-week MBITE experience. During the six-week program, 35 participants were excluded or withdrew, including 18 with incomplete attendance, 8 who withdrew consent, and 9 with incomplete post-experience questionnaire data. The final analytic sample consisted of 457 adolescents who completed all six scheduled MBITE sessions and valid post-experience questionnaire (see **Table 3**).

**Table 2:** Participant recruitment and screening flow.

Stage	n	Reason/note
Initially approached	560	Students from selected schools and classes
Assessed for eligibility	560	Screening during recruitment stage
Excluded before enrollment	68	/
Did not meet age or enrollment criteria	28	Outside eligible age range or not full-time enrolled
Parent-reported or school-recorded psychiatric diagnosis/current treatment	16	Excluded to avoid clinical confounding
Declined participation or no parental consent	24	Consent/assent not obtained
Enrolled in six-week MBITE experience	492	Provided assent and parental consent
Excluded or withdrew during the six-week program	35	/
Incomplete attendance	18	Missed one or more scheduled MBITE sessions
Withdrew consent	8	Voluntary withdrawal
Incomplete post-experience questionnaire	9	Missing key study variables
Final analytic sample	457	Completed all six MBITE sessions and valid post-experience questionnaire

**Table 3:** Information on respondents (n=457).

Categories	Options	Frequency	Percentage
Gender	Male	225	49.234%
	Female	232	50.766%
Age	10	47	10.284%
	11	49	10.722%
	12	45	9.847%
	13	48	10.503%
	14	48	10.503%
	15	51	11.160%
	16	55	12.035%
	17	52	11.379%
	18	62	13.567%
Grade level	Primary school	139	30.416%
	Junior high school	155	33.917%
	High school	163	35.667%
Type of school	Public	237	51.860%
	Private	220	48.140%
Have a religion belief?	Yes	131	28.665%
	No	326	71.335%

### 3.5 Ethical considerations

This study was conducted in full compliance with ethical guidelines for research involving minors. Informed consent was obtained from both the participants and their parents or guardians prior to participation. Additionally, participants were assured of their confidentiality and the voluntary nature of their involvement. This study received ethical approval from the authors’ institution, with identifying information anonymized.

## 4 Empirical test

### 4.1 Data screening and statistical analysis

Before the main analyses, the data were screened to assess their suitability for exploratory factor analysis, confirmatory factor analysis, and structural equation modeling. The analyses were performed using SPSS Statistics 31 and AMOS 29. Because all measurement items were assessed using five-point Likert-type scales, the item responses were treated as approximately continuous variables in the subsequent analyses. The measurement and structural models were estimated using the maximum likelihood method.

Missing data were examined before the main analyses. No missing values were identified again in the final dataset after the initial screening and exclusion of incomplete questionnaires; therefore, no imputation or listwise deletion was required. Potential outliers were assessed using standardized scores and Mahalanobis distance. No influential outliers were identified, and no additional cases were removed prior to analysis.

The normality test results indicated that the selected dataset yielded a skewness range of -0.810 to 0.734 and a kurtosis range of -0.563 to 1.077, suggesting that the data met the acceptable criteria for univariate normality and were suitable for further structural equation modeling analyses. Based on Kline’s criterion for sample size in structural equation modeling research, the final sample size of 457 was substantially larger than the minimum requirement of 140 (104). Based on commonly cited sample-size recommendations for structural equation modeling, the final sample size of 457 was considered adequate given the model complexity and the 15 observed measurement indicators. Specifically, the sample-to-indicator ratio was approximately 30:1, exceeding the commonly recommended minimum ratios of 10:1 to 20:1 (105). Consequently, the collected data were considered sufficient to address the research questions and test the proposed hypotheses.

### 4.2 Fitting test

The results of the questionnaire were analyzed in this study utilizing the AMOS 29 software via the structural equation modeling technique. The results show that CMIN/DF:1.526; CFI: 0.984; NFI: 0.954; RMSEA: 0.033; GFI: 0.966; AGFI: 0.951; TLI: 0.979. Based on the indicators from the fit test, it is evident that all fit indices for the structural equation fall within the acceptable limits of the evaluation criteria (106). As a result, the research model demonstrates a satisfactory fit.

### 4.3 Common method bias test

The findings from Podsakoff et al. (107) are utilized in this research, which incorporates Harman’s single-factor test methodology. The initial unrotated factors demonstrate that 29.270% of the variance is explained, falling short of the important threshold of 50.000%. Consequently, this study does not face challenges associated with common method bias.

### 4.4 Exploratory factor analysis

Using principal component analysis, an exploratory factor analysis was performed on the scales utilized in this research. First, the factors were rotated using the Varimax method, and items with factor loadings below 0.40 were excluded. The analysis yielded a Kaiser–Meyer–Olkin (KMO) value of 0.792, exceeding the recommended threshold of 0.70. In addition, Bartlett’s test of sphericity was statistically significant, with a chi-square value of 2,732.900, and a significance level of p < 0.05. The outcomes of the exploratory factor analysis are shown in **Table 4**, revealing that all factor loadings for the measurement items exceeded 0.600 (108). Consequently, these results suggest that the exploratory factor analysis produced favorable results, outlining the number, arrangement, and connections of the underlying factors. Therefore, no items or sample size were removed during the process.

**Table 4:** Results of exploratory factor analysis.

Factors	Measurement items	Factor loadings	Eigenvalues	EVA	ECVA
Cognitive Restructuring (CR)	CR1	0.871	2.238	14.917	14.917
	CR2	0.843			
	CR3	0.810			
Perceived Alleviation of Anxiety Symptoms (PAAS)	PAAS1	0.807	2.230	14.869	29.786
	PAAS2	0.847			
	PAAS3	0.808			
Self-acceptance (SA)	SA1	0.781	2.188	14.584	44.369
	SA2	0.872			
	SA3	0.835			
Self-efficacy (SE)	SE1	0.827	2.155	14.365	58.734
	SE2	0.818			
	SE3	0.813			
Music-Based Immersive Technology Experience (MBITE)	MBITE1	0.804	2.066	13.771	72.505
	MBITE2	0.818			
	MBITE3	0.803			

EVA is explaining the variation amount/%; ECVA is explain the cumulative variation amount/%.

### 4.5 Reliability and validity, differential validity tests

**Table 5** presents the metrics for Cronbach’s alpha, composite reliability (CR), average variance extracted (AVE), the square root of AVE, and the relationships among the latent variables. A satisfactory level of internal consistency is achieved when both Cronbach’s alpha and CR are a minimum of 0.7 (109). After confirming internal consistency, it is observed that all latent variables demonstrate a CR greater than 0.7.

**Table 5:** Results of reliability and validity tests.

Factors	Measurements	Unstd.	S.E.	Z	Std.	Cronbach’s α	AVE	CR
Music-Based Immersive Technology Experience (MBITE)	MBITE1	1.000			0.759	0.762	0.521	0.765
	MBITE2	1.066	0.088	12.125	0.726			
	MBITE3	1.028	0.087	11.844	0.678			
Cognitive Restructuring (CR)	CR1	1.000			0.819	0.823	0.611	0.825
	CR2	0.911	0.057	15.896	0.795			
	CR3	0.801	0.053	15.095	0.729			
Self-acceptance (SA)	SA1	1.000			0.684	0.803	0.582	0.806
	SA2	1.200	0.088	13.626	0.801			
	SA3	1.227	0.090	13.618	0.798			
Self-efficacy (SE)	SE1	1.000			0.769	0.789	0.557	0.791
	SE2	1.041	0.078	13.401	0.726			
	SE3	1.029	0.076	13.543	0.744			
Perceived Alleviation of Anxiety Symptoms (PAAS)	PAAS1	1.234	0.060	20.529	0.864	0.842	0.657	0.850
	PAAS2	0.881	0.055	16.100	0.687			
	PAAS3	1.000			0.867			

Moreover, the Average variance extracted (AVE) for each latent variable surpasses the accepted threshold of 0.5, suggesting a robust level of convergent validity (110, 111). Given that the square root of the AVE for these latent constructs exceeds the correlation coefficients among the various latent variables (112), it can be concluded that the discriminant validity is also adequate (refer to **Table 6**). In conclusion, the framework put forth displays sufficient internal consistency and reliability, as well as both convergent and discriminant validity. No measurement items were removed during the reliability and validity analyses because all retained items met the prespecified reliability and validity criteria.

**Table 6:** Results of differential validity test.

	Perceived alleviation of anxiety symptoms (PAAS)	Self-efficacy (SE)	Self-acceptance (SA)	Cognitive restructuring (CR)	Music-based immersive technology experience (MBITE)
Perceived Alleviation of Anxiety Symptoms (PAAS)	0.810	—	—	—	—
Self-efficacy (SE)	0.479	0.747	—	—	—
Self-acceptance (SA)	0.409	0.200	0.763	—	—
Cognitive Restructuring (CR)	0.400	0.240	0.142	0.782	—
Music-Based Immersive Technology Experience (MBITE)	0.345	0.213	0.271	0.270	0.722

### 4.6 Research hypotheses test

The structural-path analysis used the same sample of 457 participants as the analysis above. The findings from the path analysis can be found in **Table 7**. This data indicates a significant positive correlation across all influence pathways, as demonstrated by Z-values that go beyond 1.96 and p-values that are below 0.05 (109, 113).

**Table 7:** Direct path test results.

Research hypotheses	Effect paths	Unstd.	S.E.	Z	P	Std.	Results
H1	Music-Based Immersive Technology Experience (MBITE)→Perceived Alleviation of Anxiety Symptoms (PAAS)	0.092	0.041	2.246	**	0.125	Positive influence
H2	Music-based immersive technology experience (MBITE)→Cognitive Restructuring (CR)	0.342	0.070	4.881	***	0.285	Positive influence
H3	Cognitive Restructuring (CR)→Perceived Alleviation of Anxiety Symptoms (PAAS)	0.160	0.030	5.270	***	0.263	Positive influence
H5	Music-based immersive technology experience (MBITE)→Self-acceptance (SA)	0.258	0.054	4.736	***	0.284	Positive influence
H6	Self-acceptance (SA)→Perceived Alleviation of Anxiety Symptoms (PAAS)	0.232	0.042	5.543	***	0.287	Positive influence
H8	Music-based immersive technology experience (MBITE)→Self-efficacy (SE)	0.234	0.059	3.960	***	0.236	Positive influence
H9	Self-efficacy (SE)→Perceived Alleviation of Anxiety Symptoms (PAAS)	0.262	0.039	6.798	***	0.356	Positive influence

**p < 0.05; ***p < 0.01.

To examine the mediating effect, this study employed the PROCESS macro (version 4.0) with a bootstrapping procedure. A 95% confidence interval was specified, and 1,000 bootstrap resamples were generated for analysis. The results illustrating the mediating effect pathways are presented in **Table 8**. Following the procedures specified by Hayes (114), the bootstrap method was used to evaluate the significance of these mediating effects. According to the evaluation criteria—namely, a Z-value exceeding 1.96, and confidence intervals that exclude zero—it is determined that all mediating pathways are statistically significant (115).

**Table 8:** Mediation effect test results.

Hypotheses	Effect paths	SE	Z	Bias-corrected 95% CI	Percentile 95% CI
H4	Music-based immersive technology experience (MBITE)→Cognitive Restructuring (CR)→Perceived Alleviation of Anxiety Symptoms (PAAS)	0.015	3.667	(0.029, 0.090)	(0.028, 0.088)
H7	Music-based immersive technology experience (MBITE)→Self-acceptance (SA)→ Perceived Alleviation of Anxiety Symptoms (PAAS)	0.013	3.385	(0.023, 0.094)	(0.020, 0.082)
H10	Music-based immersive technology experience (MBITE)→Self-efficacy (SE)→Perceived Alleviation of Anxiety Symptoms (PAAS)	0.019	3.211	(0.028, 0.107)	(0.025, 0.101)

## 5 Conclusion and discussion

### 5.1 Findings

Using data from 457 Chinese adolescents and structural equation modeling (SEM), the study found that music-based immersive technology experience (MBITE) was significantly and positively associated with perceived alleviation of anxiety symptoms (PAAS). MBITE was also positively associated with cognitive restructuring (CR), self-acceptance (SA), and self-efficacy (SE). In turn, CR, SA, and SE were positively associated with the PAAS. The mediation results indicated that CR, SA, and SE significantly served as statistical mediators in the association between MBITE and PAAS. These findings suggest that adolescents’ perceived engagement with MBITE may be linked to PAAS through cognitive and self-related psychological resources.

### 5.2 Theoretical contributions

The theoretical contributions of this study should be understood as extensions of the hypothesized relationships derived from existing theoretical perspectives. Specifically, the hypothesized research model was informed by theories of presence and immersive engagement in virtual environments, which explain how immersive experiences may enhance attention, emotional involvement, and perceived psychological comfort (41–43), as well as music-related physiological perspectives suggesting that music may influence relaxation and stress responses through the autonomic nervous system (45, 46). The model was also grounded in cognitive-behavioral and cognitive theories of anxiety, which emphasize the role of maladaptive thoughts, cognitive restructuring, and reappraisal in anxiety-related experiences (47, 53), together with cognitive-emotional integration and dual-process perspectives that explain how emotionally engaging immersive experiences may activate both intuitive and reflective forms of processing (48, 49). In relation to self-acceptance, the model drew on self-concept and self-acceptance perspectives, which highlight the importance of congruence between the actual self and ideal self in adolescent psychological adjustment (63, 71), as well as self-determination and reward/emotional-regulation perspectives that emphasize agency, intrinsic motivation, emotional awareness, and the regulation of affective experiences (68, 69, 79). This study also incorporated self-efficacy theory, which emphasizes perceived competence, mastery, and confidence in managing challenges as important psychological resources for reducing anxiety-related distress.

This study makes several theoretical contributions to the understanding of the relationships among music-based immersive technology experience (MBITE), adolescent perceived anxiety symptoms, and related psychological factors. First, the study extends existing research on music therapy and immersive technology by examining their integration within the context of adolescent mental health. Previous studies on music therapy have primarily focused on traditional auditory, relational, or clinical settings, while immersive technology has often been applied in educational, cognitive, or simulation-based contexts (15). By examining MBITE as a culturally and technologically enriched experience, this study broadens the theoretical discussion on how multisensory music-based experiences may be associated with adolescents’ perceived emotional and psychological states.

Rather than demonstrating clinical treatment effects, the study provides exploratory evidence that adolescents’ engagement with MBITE is positively associated with perceived alleviation of anxiety symptoms (PAAS). This finding contributes to the theoretical understanding of how immersive and music-based experiences may be linked to perceived anxiety-related outcomes. In particular, the integration of music and immersive technology suggests a possible multisensory framework through which emotional engagement, attentional immersion, and perceived psychological comfort may be examined in adolescent populations (81, 116).

Second, this research contributes to the theoretical understanding of cognitive restructuring (CR), self-acceptance (SA), and self-efficacy (SE) in relation to adolescent perceived anxiety symptoms. The findings suggest that MBITE is positively associated with these psychological constructs, indicating that adolescents who report higher levels of engagement with MBITE also tend to report higher levels of CR, SA, and SE. This contributes to existing models of adolescent mental health by highlighting the potential importance of cognitive and self-related psychological resources in understanding perceived anxiety-related experiences (117).

Third, this study provides theoretical insight into the statistical mediating associations of CR, SA, and SE in the relationship between MBITE and PAAS. The mediation results suggest that these psychological constructs statistically mediate the association between MBITE and perceived alleviation of anxiety symptoms. This finding advances the theoretical understanding of possible psychological association linking immersive music-based experiences with perceived anxiety-related outcomes. Rather than positioning MBITE as a direct clinical intervention, the study suggests that CR, SA, and SE may identify psychological resources that are statistically associated with both adolescents’ engagement with MBITE and PAAS.

Moreover, this study contributes to the literature by integrating two related but previously less-connected areas: music and immersive technology. The combination of immersive environments with music-based experiences offers a useful theoretical lens for understanding how multisensory contexts may shape adolescents’ emotional engagement, self-reflection, and perceived psychological adjustment. This integration expands current conceptual models by emphasizing the role of technology-supported music experiences in adolescent mental health research.

In addition, this study contributes to the development of culturally and technologically contextualized perspectives on adolescent mental health. By examining MBITE within a Chinese adolescent sample, the study highlights the importance of cultural relevance, sensory immersion, and individual psychological resources in understanding perceived anxiety-related experiences (118, 119). This provides a theoretical basis for future research to further investigate how culturally adapted and technology-enhanced music experiences may be associated with adolescent psychological well-being.

In conclusion, the theoretical contributions of this study lie in its integration of MBITE with key psychological constructs, including CR, SA, SE, and PAAS. The findings provide exploratory evidence of statistical associations among these variables and offer a possible theoretical framework for understanding the psychological association through which immersive music-based experiences may relate to perceived anxiety symptoms among adolescents.

### 5.3 Practical contributions

The findings of this study provide valuable practical insights for adolescent mental health support, educational practice, and the design of technology-enhanced music-based activities. Given the observational and exploratory nature of the study, these implications should be understood as preliminary and supportive rather than as evidence of clinical treatment effectiveness.

The implementation and accessibility of immersive technology should also be considered when interpreting the practical implications of MBITE. Although immersive environments can present synchronized auditory and visual stimuli in a controlled digital setting, their use requires appropriate hardware, software, technical support, space management, equipment hygiene procedures, and supervision. These requirements may affect the feasibility of implementing MBITE in schools, community settings, or lower-resource environments. In addition, the cost of VR headsets, software maintenance, content development, and staff training may create unequal opportunities for implementation across regions or institutions. Some adolescents may also experience cybersickness, visual fatigue, discomfort, or low acceptance of head-mounted devices. Therefore, the findings should be interpreted within the specific technological and institutional context of this study and should not be generalized uncritically to all forms of music-based support or immersive technology. Accordingly, future applications of MBITE should consider not only its psychological and experiential potential but also the technical, institutional, financial, and user-acceptance conditions required for responsible implementation.

First, this study provides practical insight into the potential value of music-based immersive technology experience as a supportive and experience-based approach in the context of adolescent perceived anxiety-related symptoms. The results showed significant positive associations between music-based immersive technology experience and perceived alleviation of anxiety symptoms, cognitive restructuring, self-acceptance, and self-efficacy. In addition, significant positive associations were also observed between cognitive restructuring, self-acceptance, self-efficacy, and perceived alleviation of anxiety symptoms. These findings suggest that music-based immersive technology experience is not only statistically related to perceived anxiety alleviation but also associated with psychological resources that are positively related to perceived anxiety alleviation. Such associations may provide useful reference points for school counselors, educators, and practitioners involved in school-based mental health support when designing non-clinical activities aimed at supporting adolescents’ emotional awareness, cognitive flexibility, self-acceptance, and confidence in managing challenges.

Second, in educational settings, the findings may inform the development of school-based mental health support programs. Schools may consider incorporating music-based and immersive digital experiences into broader well-being activities, psychological education programs, or extracurricular mental health initiatives. Such activities may provide engaging and interactive contexts in which students can reflect on their emotions, develop more adaptive ways of thinking, strengthen self-understanding, and build confidence in coping with difficulties. However, these applications should be implemented as supportive or preventive activities rather than as substitutes for professional psychological or psychiatric treatment.

Third, this study offers practical implications for the design and management of adolescent mental health programs. Program developers and administrators in schools, community youth centers, and non-clinical mental health service settings may use these findings to consider how immersive technologies can be integrated with music-based experiences in a developmentally appropriate and culturally sensitive manner. The significant positive associations between cognitive restructuring, self-acceptance, self-efficacy, and perceived alleviation of anxiety symptoms suggest that practical programs may benefit from incorporating components related to cognitive adjustment, self-reflection, self-acceptance, and confidence-building. Therefore, adolescent mental health support programs should not focus solely on perceived symptom alleviation but may also consider strengthening psychological resources that are positively associated with perceived anxiety alleviation.

In addition, the findings may be useful for practitioners involved in adolescent counseling, psychological education, and well-being promotion. Although this study does not establish clinical efficacy, it highlights the relevance of cognitive restructuring, self-acceptance, and self-efficacy as psychological resources statistically associated with perceived alleviation of anxiety symptoms. Practitioners may therefore consider incorporating activities that encourage adolescents to reframe negative thoughts, develop a more accepting attitude toward themselves, and strengthen their perceived ability to cope with challenges when designing music-based or technology-supported mental health education activities.

At the policy and program planning level, this study provides preliminary evidence that may inform future discussions on technology-supported adolescent mental health promotion. Policymakers and educational administrators may consider supporting exploratory pilot programs that integrate digital technology, music-based engagement, and psychological education in school or community settings. Such initiatives may contribute to more accessible and engaging mental health support systems for adolescents, particularly when implemented alongside appropriate ethical safeguards, professional supervision, and referral mechanisms for students requiring clinical care. The observed positive associations among music-based immersive technology experience, cognitive restructuring, self-acceptance, self-efficacy, and perceived alleviation of anxiety symptoms may also help guide the design of future programs that combine emotional support with the cultivation of adaptive psychological resources.

In conclusion, the practical contributions of this study lie in its potential to inform the design of supportive, non-clinical, and technology-enhanced music-based mental health activities for adolescents. These results offer useful insights for educators, counselors, program developers, and policymakers by highlighting the importance of both immersive music-based experiences and related psychological resources in adolescent mental health support. These findings may also provide a basis for future pilot interventions, longitudinal research, and evidence-based program development.

## 6 Research limitations

While this study provides valuable empirical evidence regarding the associations among music-based immersive technology experience (MBITE), perceived alleviation of anxiety symptoms (PAAS), cognitive restructuring (CR), self-acceptance (SA), and self-efficacy (SE), several limitations should be acknowledged.

First, the dependent construct measured adolescents’ perceived alleviation of anxiety symptoms rather than clinical anxiety severity, diagnostic status, or objectively verified symptom change. Although the PAAS items captured perceived reductions in anxiety-related physical discomfort, repetitive or intrusive anxious thoughts, and emotional uneasiness, they should not be interpreted as a substitute for validated anxiety severity measures. Therefore, the findings should be understood as evidence regarding adolescents’ subjective perception of anxiety-related relief, not as evidence of clinically confirmed anxiety reduction. Therefore, the findings should not be generalized to clinical populations without further research using professional diagnostic assessments, clinical samples, and validated anxiety severity instruments.

Second, this study employed an observational and cross-sectional design, which limits the ability to infer causality or examine changes over time. Although structural equation modeling strengthened the analytical rigor, the absence of longitudinal data prevents conclusions about temporal ordering or the stability of the observed associations. Future research should adopt longitudinal or experimental designs to examine how perceived anxiety-related symptoms and related psychological constructs may change over time.

Third, the sample consisted of 457 adolescents from several schools in China, which may limit the generalizability of the findings. Cultural, socio-economic, and educational factors may influence adolescents’ anxiety-related experiences, engagement with music-based activities, and perceptions of immersive technologies. Future studies should replicate the research across diverse cultural, regional, and demographic contexts.

Fourth, although this study focused on cognitive restructuring, self-acceptance, and self-efficacy as mediating variables, other relevant factors were not examined. Variables such as personality traits, family support, digital literacy, baseline mental health status, and prior exposure to music-based or immersive experiences may also shape the associations among MBITE, PAAS, CR, SA, and SE. Future research should include broader psychological and contextual variables.

Fifth, this study relied primarily on self-reported questionnaire data, and all variables were collected at a single time point, which may be affected by social desirability bias, recall bias, subjective misreporting, or common method variance. Although Harman’s single-factor test was conducted, this test provides only a preliminary assessment and cannot fully rule out common method bias. Accordingly, the observed associations in this study should be interpreted with caution. Future studies could combine self-report measures with qualitative interviews, behavioral observations, physiological indicators such as heart rate variability and cortisol levels, standardized anxiety measures, and, where appropriate, clinician-rated, parent-reported, or teacher-reported indicators to enhance the robustness and validity of the findings. Future research could also reduce common method bias by collecting data from multiple sources or at different time points.

Another limitation concerns the technological and institutional context in which MBITE was implemented. The feasibility and accessibility of immersive technology may vary depending on hardware availability, technical support, cost, space management, supervision, and adolescents’ acceptance of head-mounted devices. Therefore, the findings should be interpreted within the specific implementation context of this study and should not be generalized uncritically to all schools, community settings, or lower-resource environments. Future research should compare different hardware platforms, evaluate lower-cost or non-headset formats, and examine feasibility across schools with different levels of resources.

Seventh, the cultural specificity of the Chinese traditional music materials used in MBITE. The emotional and reflective responses observed in this study may have been influenced by Chinese adolescents’ familiarity with these musical elements and the cultural meanings attached to them. As a result, the findings should not be assumed to apply equally to adolescents from other cultural backgrounds or to immersive music experiences based on other musical traditions. Future studies could examine whether similar associations are observed when MBITE is adapted with culturally relevant music materials for adolescents in different cultural contexts.

Final, although the measurement items used in this study were selected and adapted from mature and validated scales, the study did not administer the complete original instruments or established short-form scales for all constructs. This decision was made to balance construct coverage, contextual relevance, and respondent burden among adolescent participants. However, selecting particular items from different scales may limit the content validity and comparability of the measures. Future research should consider using validated short-form instruments or conducting more comprehensive scale validation to further confirm the robustness of the measurement model.

## Data Availability

The data presented in this study are available from the corresponding author upon reasonable request, subject to ethical restrictions and participant privacy protections. Because the study involved adolescent participants, raw participant-level data cannot be made publicly available without restriction. To support transparency and reproducibility, the authors can provide de-identified analysis data where ethically permissible, together with relevant analysis settings. Requests to access the datasets should be directed to ZhaoLei Sun. szl@kangwon.ac.kr.
