# Supplementary material for: Music-based immersive technology experience and adolescents’ perceived alleviation of anxiety symptoms: cross-sectional evidence on the mediating roles of cognitive restructuring, self-acceptance, and self-efficacy
Source: Front Psychiatry. 2026 Oct 19;17:1917295. doi: 10.3389/fpsyt.2026.1917295 (PMC13725355; doi:10.3389/fpsyt.2026.1917295)
Supplement: Supplementary file 1 [file DataSheet1.docx]

**Appendix A. Session-by-session MBITE protocol**

| **Session** | **Music track / version / source** | **Visual scene** | **Duration and sequence** | **Guided verbal prompts** | **Participant interaction** |
| --- | --- | --- | --- | --- | --- |
| Week 1 | Guqin excerpt adapted from *Flowing Water*; professionally recorded instrumental version; same edited audio file used for all participants | Mountain stream, slow-moving clouds, still water, distant rocks | Orientation and comfort check 3-5 min; attentional settling 3-5 min; main music-VR segment 20-25 min; guided prompts 3-5 min distributed across the session; closing transition 2-3 min | “Notice your breathing and let your attention settle with the music.” “Observe the sound and scene without forcing anything.” “Allow your body to become still and comfortable.” | Seated viewing only; natural head movement allowed; no task response; participants could request to pause or stop |
| Week 2 | Bamboo flute excerpt adapted from *Autumn Moon over the Calm Lake*; professionally recorded instrumental version; same edited audio file used for all participants | Calm lake, moonlight reflection, soft ripples, open sky | Same standardized session structure as Week 1 | “Observe any worrying thought as it comes and goes.” “See whether you can watch the scene without judging yourself.” “Let the music carry your attention back to the present moment.” | Seated viewing only; natural head movement allowed; no task response; participants could request to pause or stop |
| Week 3 | Erhu excerpt adapted from *Fisherman’s Evening Song*; professionally recorded instrumental version; same edited audio file used for all participants | Riverboat, sunset, distant shoreline, warm low light | Same standardized session structure as Week 1 | “Try to look at one difficulty from another angle.” “Notice whether the difficulty feels different when viewed from a distance.” “Allow another possible explanation to appear.” | Seated viewing only; natural head movement allowed; no task response; participants could request to pause or stop |
| Week 4 | Guqin excerpt adapted from *Three Variations on the Plum Blossom*; professionally recorded instrumental version; same edited audio file used for all participants | Plum blossoms, winter branches, light snow, pale background | Same standardized session structure as Week 1 | “Acknowledge the parts of yourself that are imperfect without criticism.” “Allow difficulty and growth to exist together.” “Notice one quality in yourself that you can accept today.” | Seated viewing only; natural head movement allowed; no task response; participants could request to pause or stop |
| Week 5 | Chinese instrumental ensemble excerpt adapted from *Spring River Flowers by Moonlight*; professionally recorded instrumental version; same edited audio file used for all participants | Spring river, drifting petals, reflected lights, gentle movement | Same standardized session structure as Week 1 | “Recall one challenge you have managed before.” “Notice your ability to continue when something feels hard.” “Let this memory remind you that you have handled difficulties before.” | Seated viewing only; natural head movement allowed; no task response; participants could request to pause or stop |
| Week 6 | Bamboo flute and ensemble excerpt adapted from *Birds in the Deep Mountains*; professionally recorded instrumental version; same edited audio file used for all participants | Forest path, moving light, open valley, distant birds | Same standardized session structure as Week 1 | “Bring together what you have noticed across the sessions.” “As the session ends, take one calm thought or feeling with you.” “Notice that you can return to steadiness after tension.” | Seated viewing only; natural head movement allowed; no task response; participants could request to pause or stop |

*Note: Across weeks, the protocol structure was identical; only the themed music excerpt and corresponding visual scene varied. All audio files were edited to a comparable duration and loudness level before implementation. The complete audiovisual sequence, prompt timing, and session order were embedded in the Unity-based software and were identical for all participants within each weekly session. No music track, visual scene, guided prompt, or presentation sequence was modified after the main six-week MBITE program began.*

**Appendix B. Chinese-language questionnaire items and scoring instructions**

This Appendix B provides the Chinese-language measurement items actually administered to participants. Participants completed the questionnaire in Chinese after finishing the MBITE experience. All items were rated on a five-point Likert scale: 1 = 非常不同意, 2 = 不同意, 3 = 不确定, 4 = 同意, and 5 = 非常同意. Scores for each construct were calculated by averaging the three corresponding items. Higher scores indicated higher levels of the corresponding construct. No items were reverse-coded. PAAS items assessed participants’ retrospective self-reported perception of anxiety-related relief and were not intended to measure clinical anxiety severity, diagnostic status, or objectively verified symptom reduction.

| **Construct** | **Chinese construct label** | **Item code** | **Chinese-language item administered to participants** | **Scoring** |
| --- | --- | --- | --- | --- |
| Music-Based Immersive Technology Experience (MBITE) | 基于音乐的沉浸式技术体验 | MBITE1 | 当我参与基于音乐的沉浸式技术体验时，我有一种沉浸其中的感觉，好像自己进入了另一个空间。 | 1-5; higher scores indicate stronger perceived MBITE |
| Music-Based Immersive Technology Experience (MBITE) | 基于音乐的沉浸式技术体验 | MBITE2 | 当我参与基于音乐的沉浸式技术体验时，我感觉自己、音乐和虚拟环境之间有较紧密的联系。 | 1-5; higher scores indicate stronger perceived MBITE |
| Music-Based Immersive Technology Experience (MBITE) | 基于音乐的沉浸式技术体验 | MBITE3 | 当我参与基于音乐的沉浸式技术体验时，我经常会深深投入其中。 | 1-5; higher scores indicate stronger perceived MBITE |
| Cognitive Restructuring (CR) | 认知重构 | CR1 | 我能够从不同角度看待让我困扰的问题。 | 1-5; higher scores indicate stronger cognitive restructuring |
| Cognitive Restructuring (CR) | 认知重构 | CR2 | 当遇到具有挑战性的情况时，我能够调整自己的看法，以减少负面情绪。 | 1-5; higher scores indicate stronger cognitive restructuring |
| Cognitive Restructuring (CR) | 认知重构 | CR3 | 即使在困难的情况下，我也能够发现其中积极的一面。 | 1-5; higher scores indicate stronger cognitive restructuring |
| Self-acceptance (SA) | 自我接纳 | SA1 | 我正在逐渐学会接纳自己的不足。 | 1-5; higher scores indicate stronger self-acceptance |
| Self-acceptance (SA) | 自我接纳 | SA2 | 我能够欣赏自己独特的地方。 | 1-5; higher scores indicate stronger self-acceptance |
| Self-acceptance (SA) | 自我接纳 | SA3 | 我能够以较宽容的态度看待自己的缺点。 | 1-5; higher scores indicate stronger self-acceptance |
| Self-efficacy (SE) | 自我效能感 | SE1 | 我相信自己有能力面对生活中的各种挑战。 | 1-5; higher scores indicate stronger self-efficacy |
| Self-efficacy (SE) | 自我效能感 | SE2 | 即使遇到困难，我也有信心坚持下去。 | 1-5; higher scores indicate stronger self-efficacy |
| Self-efficacy (SE) | 自我效能感 | SE3 | 当问题出现时，我相信自己能够做出合适的决定。 | 1-5; higher scores indicate stronger self-efficacy |
| Perceived Alleviation of Anxiety Symptoms (PAAS) | 感知焦虑症状缓解 | PAAS1 | 我感觉与焦虑有关的身体不适有所减轻，例如心跳加快、呼吸不适或身体紧张。 | 1-5; higher scores indicate stronger perceived alleviation |
| Perceived Alleviation of Anxiety Symptoms (PAAS) | 感知焦虑症状缓解 | PAAS2 | 我感觉反复出现或侵入性的焦虑想法有所减少。 | 1-5; higher scores indicate stronger perceived alleviation |
| Perceived Alleviation of Anxiety Symptoms (PAAS) | 感知焦虑症状缓解 | PAAS3 | 我感觉日常生活中与焦虑有关的情绪不安有所减轻。 | 1-5; higher scores indicate stronger perceived alleviation |
